# The effect of low-level laser irradiation (In-Ga-Al-AsP - 660 nm) on melanoma *in vitro *and *in vivo*

**DOI:** 10.1186/1471-2407-9-404

**Published:** 2009-11-20

**Authors:** Lúcio Frigo, Juliana SS Luppi, Giovani M Favero, Durnavei A Maria, Sócrates C Penna, Jan M Bjordal, Rene J Bensadoun, Rodrigo AB Lopes-Martins

**Affiliations:** 1 Biological Sciences and Health Center, Cruzeiro do Sul University. Av. Dr. Ussiel Cirilo, 225 São Miguel Paulista, 08060-070 São Paulo, SP - Brasil; 2 State University of Ponta Grossa, General Biology Department, Av. Gal. Carlos Cavalvcanti, 4748 Ponta Grossa 84030-900, PR - Brasil; 3 Laboratory of Biochemistry and Biophysics, Butantan Institute, Av. Dr. Vital Brasil, 1500 São Paulo 05599-000, SP - Brasil; 4 Laboratory of Pharmacology and Phototherapy of Inflammation, Department of Pharmacology, Institute of Biomedical Sciences, University of São Paulo - São Paulo 05508-900 SP - Brasil; 5 Institute for Physiotherapy, Bergen University College, Moellendalsvn. 6, 5009 Bergen, Norway; 6 Section of Physiotherapy Science, Institute of Public Health and Primary Health Care, University of Bergen, Kalfarveien 31, 5018 Bergen, Norway; 7 Service d'Oncologie Radiothérapique, CHU de Poitiers, 2, rue de la Milétrie, BP 577, 86021 - Poitiers Cedex, France

## Abstract

**Background:**

It has been speculated that the biostimulatory effect of Low Level Laser Therapy could cause undesirable enhancement of tumor growth in neoplastic diseases. The aim of the present study is to analyze the behavior of melanoma cells (B16F10) *in vitro *and the *in vivo *development of melanoma in mice after laser irradiation.

**Methods:**

We performed a controlled *in vitro *study on B16F10 melanoma cells to investigate cell viability and cell cycle changes by the Tripan Blue, MTT and cell quest histogram tests at 24, 48 and 72 h post irradiation. The *in vivo *mouse model (male Balb C, n = 21) of melanoma was used to analyze tumor volume and histological characteristics. Laser irradiation was performed three times (once a day for three consecutive days) with a 660 nm 50 mW CW laser, beam spot size 2 mm^2^, irradiance 2.5 W/cm^2 ^and irradiation times of 60s (dose 150 J/cm^2^) and 420s (dose 1050 J/cm^2^) respectively.

**Results:**

There were no statistically significant differences between the *in vitro *groups, except for an increase in the hypodiploid melanoma cells (8.48 ± 1.40% and 4.26 ± 0.60%) at 72 h post-irradiation. This cancer-protective effect was not reproduced in the *in vivo *experiment where outcome measures for the 150 J/cm^2 ^dose group were not significantly different from controls. For the 1050 J/cm^2 ^dose group, there were significant increases in tumor volume, blood vessels and cell abnormalities compared to the other groups.

**Conclusion:**

LLLT Irradiation should be avoided over melanomas as the combination of high irradiance (2.5 W/cm^2^) and high dose (1050 J/cm^2^) significantly increases melanoma tumor growth *in vivo*.

## Background

Malignant melanoma represents a burden to modern society and requires considerable efforts in terms of health service utilization. The incidence is increasing worldwide and in the Netherlands the prevalence is currently 16.1/100,000 with a mortality rate of 3.0/100,000[1].

Low level laser therapy (LLLT) has gained increasing popularity as a treatment for soft tissue injuries and joint conditions. It is applied transcutaneously with typical irradiances being 10 mW/cm^2 ^- 5,000 mW/cm^2^, treatments times being in the range of 10 seconds - 2 minutes, with total energy delivered of 1 - 4 Joules(J)/cm^2 ^per point when targeting joints, tendons and muscles. The cellular proliferative potential of LLLT irradiation has attracted some negative speculation that this could also increase tumor growth in neoplasic diseases. Previous studies of LLLT irradiation of tumor cells in vitro have generated conflicting research data across a range of cultivated tumor cell lines and irradiation parameters [2-11] but there have been relatively few *in vivo *studies published [12,13]. *In vivo *studies are essential for the study of disease development and should be the main tool for studying the behavior of tumor cells. The complexity of the multi-cellular environment in an ongoing disease makes it hard to predict tumor behavior and cell culture studies alone are inadequate to for assessment of tumor responses.

Increases in cell proliferation and collagen biosynthesis after LLLT in wound healing improvement has already been observed in the pioneer work of Mester et al. [14]. The following decades were marked by a large quantity of research articles in LLLT. A better understanding of laser light modulatory mechanisms was obtained, but this effort also yielded conflicting results. There is a shortage of evidence about the effects of LLLT in malignant conditions such as melanoma. The complete biochemical mechanisms of cell proliferation after LLLT irradiation are still uncertain and we believe there is a need to study the effects of LLLT on tumor growth in suitable cell and animal models.

The aim of the present work is to study the effect of LLLT irradiation both *in vitro *and *in vivo*. For this purpose we decided to study cell viability and cell cycle changes in melanoma cells (B16F10) *in vitro*, and their behavior when injected subcutaneously into Balb C mice *in vivo*.

## Methods

All the experimental procedures were submitted to and approved by the Ethical Committee at the Cruzeiro do Sul University.

### Cell culture

B16F10 murine melanoma cells were obtained from ATCC (clone CRL 6457). Melanoma cells were grown in RPMI 1640 medium supplemented with 10% fetal bovine serum (FBS), 100 U/mL penicillin/streptomycin and 24 mM NaHCO_3 _at 37°C in a humidified atmosphere containing 5% CO_2_. Cells were seeded at an initial density of 2 × 10^4 ^cells/cm^2 ^(B16F10) for cell viability, which was determined by the MTT method and 1 × 10^6 ^cells/cm^2 ^for the Trypan blue exclusion test.

### *In vitro *laser irradiation

B16F10 cells were irradiated a total of three times (once a day for three consecutive days) in a 96 well culture plate for the MTT method and in a 12 well plate for Trypan blue and cytometric assays. Irradiation was performed with a 660 nm, 50 mW Continuous Wave (CW) laser, beam spot size 2 mm^2^, irradiance 2.5 W/cm^2 ^(Quasar Medical - Dentoflex, São Paulo, Brasil). The seeded wells were spaced 5 cm apart in all directions and a thin aluminum sheet was placed halfway (2.5 cm) between them to prevent unintentional light scattering between the wells. The wells were randomly divided into a control group which received no irradiation, and a treatment group which received an LLLT dose of 150 J/cm^2 ^with an irradiance of 2.5 W/cm^2 ^for 60 seconds (3J), while a second group received sessions with an LLLT dose of 1050 J/cm^2 ^with an irradiance of 2.5 W/cm^2 ^for 420 seconds (21J). Total energy delivered after all three sessions was 9J and 63J respectively in the irradiated groups. A support device held the LLLT emission tip perpendicular to and 2 mm distant from the culture media. Irradiation was carefully timed and carried out in a dark laminar flux hood.

### Animals

The animals were isogenic male Balb C mice (n = 21), which were randomized into one of three groups; a control group (n = 7), a "low" dose group (n = 7) and a "high" dose group (n = 7). The mice were injected subcutaneously with a suspension of 2 × 10^6 ^B16F10 melanoma cells.

### *In vivo *laser irradiation

After fifteen days of tumor growth the animals were irradiated three times (once a day for three consecutive days) at the site of the injected melanoma cells with the same laser and laser parameters as used in the *in vitro *study. Irradiation was performed with a 660 nm 660 nm, 50 mW Continuous Wave (CW) laser, beam spot size 2 mm^2^, irradiance 2.5 W/cm^2 ^(Quasar Medical - Dentoflex, São Paulo, Brasil). Control Group: Received no irradiation Group 1: Received three LLLT sessions (once a day for three consecutive days) each of 60 seconds with a dose of 150 J/cm^2^, (energy delivered per session was 3J, total energy delivered after three sessions was 9J) Group 2: Received three sessions (once a day for three consecutive days) each of 420 seconds with a dose of 1050 J/cm^2^, (energy delivered per session was 21J, and total energy delivered after three sessions was 63J).

### Outcome measures *in vitro*

Cell viability and cell changes were determined by MTT method and Trypan blue exclusion tests (B16F10). Cells were seeded at a density of 1 × 10^6 ^cells/Cm^2 ^(B16F10). At the end of the experiment, cells were treated with trypsin (0.05% trypsin in 0.02% EDTA) and washed 3 times with PBS, fixed in 70% ethanol, and stained with propidium iodide (PI) 50 mg/10 uL final concentration, these can distinguish hypodiploid (non-viable or dead cells) from diploid (viable) cells, for 30 min in the dark. All analyses were done using a FACScalibur flow cytometer (Becton Dickinson, San Jose, CA). The red fluorescence of PI was collected through a 585/42-nm band-pass filter, and the fluorescence signals were measured in a linear scale of 1024 channels. For each sample, at least 10,000 events were acquired and the data were analyzed using appropriate software (CELLQuest, Becton Dickinson, San Jose, CA). Cell viability was assessed by counting adherent and non-adherent cells and measured by the cellular permeability to propidium iodide. Cells in S/G_2_/M (proliferating) and G_0_/G_1 _phases, and hypodiploid cells (cells under death process) were analyzed.

### Outcome measures *in vivo*

Tumor cell growth area was estimated measuring length and width with a paquimeter device and using the formula: volume = length × width^2 ^π div 6. Histological tumor analysis was performed after tumor volume measurements. Animals were anaesthetized with inhaled halothane and sacrificed by cervical dislocation. Tumor mass was immediately removed and immersed in a 4% phosphate buffered paraformaldehide solution for 48 h. Specimens were dehydratated and embedded in paraffin prior to the 5 μm microtome sections. Histological sections were collected on glass slides and hematoxylin-eosin stained. Analysis and photographs were carried out in a Nikon-YS100 photomicroscope.

### Statistical analysis

The obtained data were first plotted for analysis of normal distribution, and statistical analysis was then performed with parametric tests if the data were normally distributed. The statistical level of significance was set at P < 0.05, and significance was tested statistically by an ANOVA-test. The mean values and its standard error (SE) were calculated, and differences between control group data and the irradiated group data were tested statistically with Bonferroni's test.

## Results

### *In vitro *experiments

**Figure 1:**
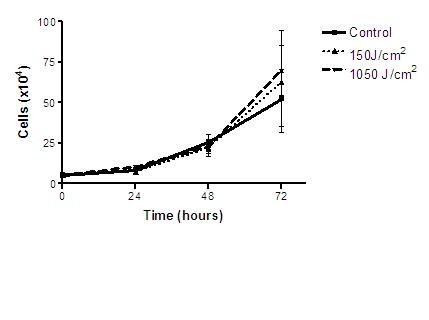
**Total B16F10 cell count number by Trypan blue exclusion method**. No significant differences were observed.

The Trypan Blue dye exclusion test showed no statistical differences in proliferation or cell death numbers among irradiated groups and control group in the different times analyzed (Figure 1).

**Figure 2:**
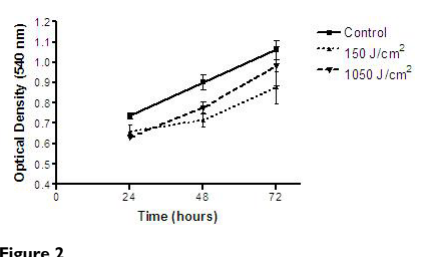
**Proliferative-inhibitory effects of low-level laser irradiation in B16F10 cells evaluated by MTT colorimetric method**. * = p < 0.05.

The MTT colorimetric test showed no statistical differences in proliferation or cell death numbers among irradiated groups and control group in the different times analyzed (Figure 2).

**Figure 3:**
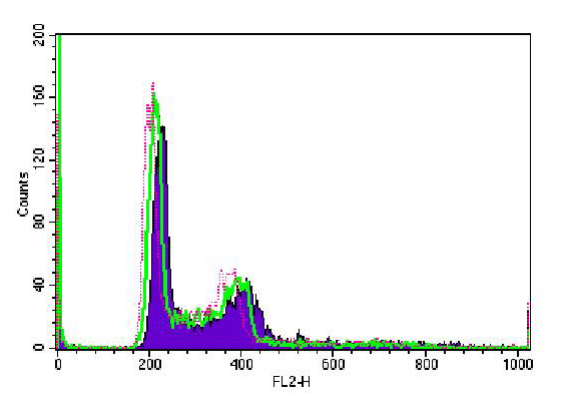
**Cell-Quest acquisition histogram of B16F10 cell cycle phases after 24 h irradiation**. Red dot refers to control group; green line refers to 150 J/cm^2 ^group and purple curve refers to 1050 J/cm^2 ^group.

**Figure 4:**
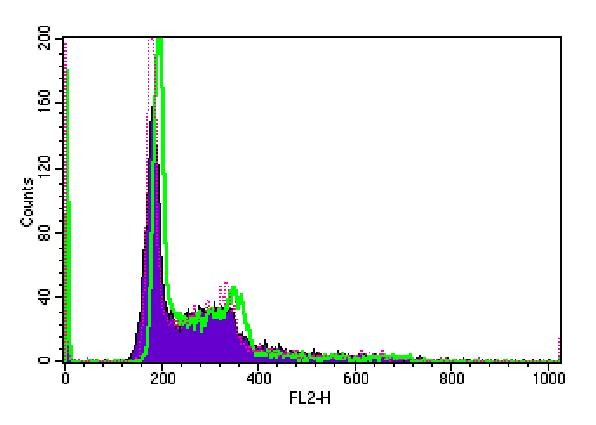
**Cell-Quest acquisition histogram of B16F10 cell cycle phases after 48 h irradiation**. Red dot refers to control group; green line refers to 150 J/cm^2 ^group and purple curve refers to 1050 J/cm^2 ^group.

**Figure 5:**
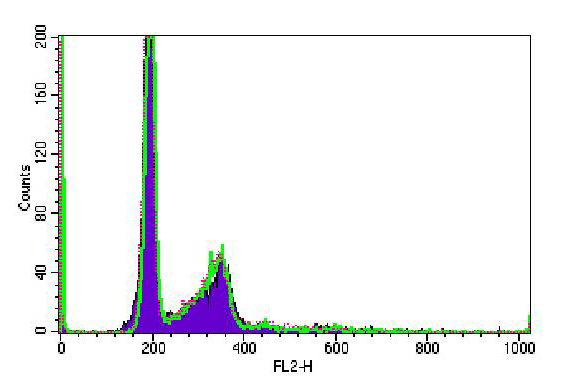
**Cell-Quest acquisition histogram of B16F10 cell cycle phases after 72 h irradiation**. Red dot refers to control group; green line refers to 150 J/cm^2 ^group and purple curve refers to 1050 J/cm^2 ^group.

Cell cycle analysis in B16F10 cells showed no statistically significant differences in the cell numbers in G0/G1, S, G2/M phases at 24 h, 48 h and 72 h among irradiated groups and control group (Figure 3, 4, 5).

**Figure 6:**
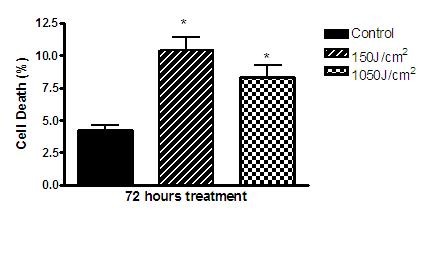
**Graphic showing B16F10 cell death percentage after 72 h of laser irradiation**. Statistically significant difference is indicated by asterisk (*P < 0,05).

There was statistically a significant difference (p < 0.05) in hypodiploid cells (possible cell death) at 72 h between the irradiated and control groups (8.48 ± 1.40% and 4.26 ± 0.60%). The increase in apoptosis was most prominent in the low dose 150 J/cm^2 ^group (Figure 6).

### *In vivo *experiments

**Figure 7:**
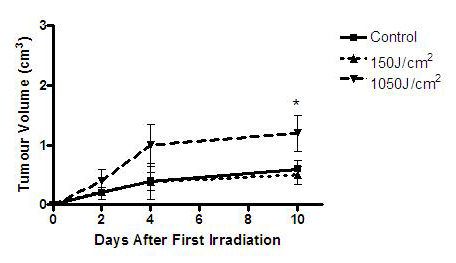
**Graphic showing melanoma tumor growth in the three groups until the 10^th ^day after irradiation**.

15 days after the B16F10 cell injections all the animals presented average tumor mass volume of 0.12 ± 0.04 cm^3^. The increase of the tumor mass volume of control and irradiated groups are shown in Figure 7.

**Figure 8:**
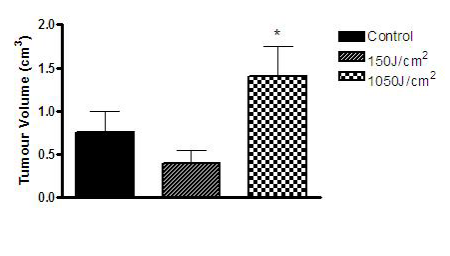
**Graphic showing the percentage of volume growth on the 10^th ^day after irradiation of the three groups**. * = p < 0.05 when compared to the control group indicated by One Way ANOVA and Bonferroni's Multiple Comparison Test (*P < 0,05).

At the 10th day, the tumor mass volume was significantly higher in the 1050 J/cm^2 ^group when compared to the 150 J/cm^2 ^and the control group. No significant difference in tumor volume was observed between the 150 J/cm^2 ^and the control group (Figure 8).

**Figure 9:**
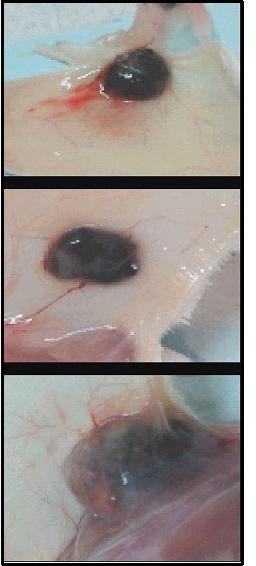
**photography showing the macroscopic appearance of the tumor**. A - control group, B - 150 J/Cm^2 ^group and C - 1050 J/Cm^2 ^group.

The macroscopic appearance of dissected tumor differed between the 1050 J/cm^2 ^group and the two other groups. In addition to a marked increase of the volume of this group, the connective tissue of the capsule appeared sticky to the tumor mass and to the adjacent muscle tissue. A greater number of blood vessels were also observed (Figure 9)

**Figure 10:**
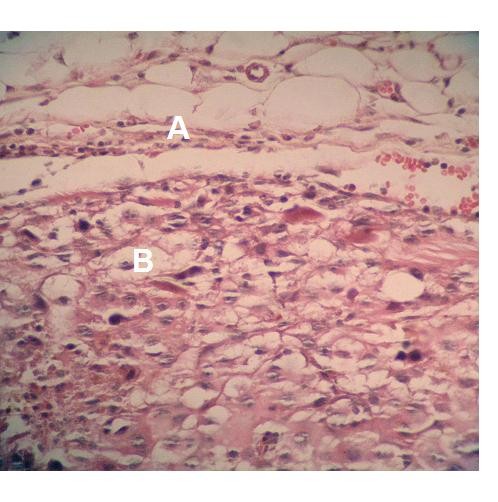
**Hematoxylin-eosin stained photomicrograph showing tumor cells (A), connective tissue tumor capsule (B) Tumor mass in the control group**. ×400.

Histological sections of the control group revealed a dense mass of melanin producing melanoma cells invaded by lymphocytes, plasma cells and macrophages. A rich vascular bed filled with leukocytes and red blood cells can be observed. Some restricted areas of necrotic tissue were also present. In the connective tissue of the capsule, immunological cells spread through thin collagen fibers and edema areas (Figure 10).

**Figure 11:**
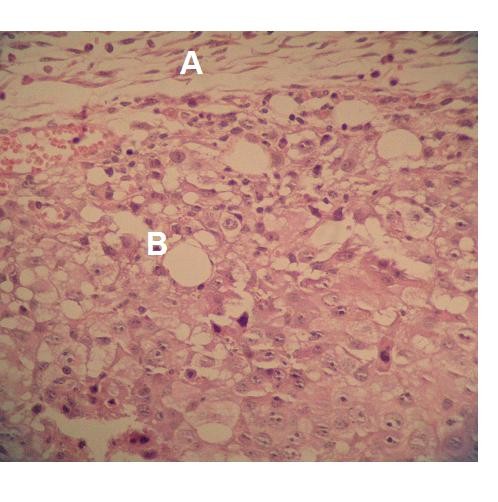
**Hematoxylin-eosin stained photomicrograph showing (A), connective tissue tumor capsule (B) tumor mass in the 150 J/Cm^2^. ×400**.

In the histological sections of the 150 J/cm^2 ^group, immune cells were less frequent in the tumor mass, and large blood vessels were filled with leukocytes and red blood cells. Necrotic areas were slightly larger compared to the control group. The connective tissue of the capsule had fewer immune cells in a greater area of thin fibers of collagen (Figure 11).

**Figure 12:**
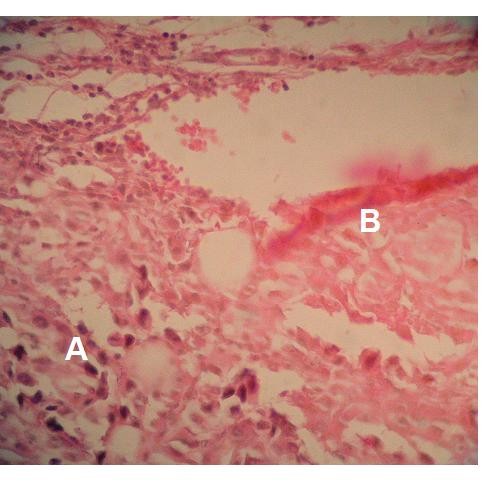
**Hematoxylin-eosin stained photomicrograph showing tumor mass (A), necrotic area (B) in the 1050 J/Cm^2^. ×400**.

Histological sections of the 1050 J/cm^2 ^group showed remarkably atypical melanoma cells. Nuclei were of various sizes and shapes, and apoptotic figures and the frequency of mitotic cells were high. Necrotic areas were more common and extensive compared to the other groups. Immune cells were observed in greater numbers in the tumor mass and in the highly vascular capsule (Figure 12).

## Discussion

In the present paper we have investigated the effects of LLLT on malignant melanoma, *in vitro *and *in vivo*. The question of a potential unwanted proliferative effect of low-level laser irradiation, has been raised by some authors [15,16]. We observed that laser irradiation with a low LLLT dose of 150 J/cm^2 ^presented opposite effects when applied to each distinct situation. In the cultured melanoma cells, we found that the two LLLT doses presented a non-significant effect on tumor cells or even an inhibitory effect of cancer cell proliferation through increased apoptosis. In the *in vivo *experiment the low dose (150 J/cm^2^) was not inducing any changes in the cancer cell behavior. However, the high dose (1050 J/cm^2^) showed a significant increase in tumor mass volume and considerable histological alterations which indicate a worsening of the cancer. The results have several implications for research and clinical practice.

Cell culture is an important method for studying basic biological processes and to understand the possible cell reactions to treatments. Many kinds of tumor cell lines have been studied, ranging from carcinomas to sarcomas and myelomas [3,4,6,8,10,17]. We chose B16F10 melanoma cell line because it's a pigmented, highly aggressive and invasive tumor [18]. Our results of non-significant LLLT effects in the *in vitro *tests of cell viability are in accordance with the largely non-significant findings of other authors [9].

Our cell cycle analysis with flow cytometry method indicated a significant increase in cell death in 72 h of the 1050 J/cm^2 ^group. Some authors have previously found increased cell death *in vitro *after LLLT irradiation. LLLT fluences higher than 6 J/cm^2 ^seemed to increase cell death in melanoma cell lines (G361, LD50 and SKmel-23), and especially in melanin producing cells [19]. There seems to be an inverse relationship between laser fluence and melanoma cell growth in culture [11]. Other authors have reported an increase in G_0_/G_1 _phase of the cell cycle using HTB66 melanoma cell line [2], but our results did not support this finding. One important aspect of our findings is the discrepancy between the *in vitro *and *in vivo *experiments. It seems necessary to be careful in generalizing *in vitro *results, as cell-matrix interactions and cell behavior in the complex environment of tissues may produce unexpected reactions.

Our results demonstrated a significant tumor growth when the animals were irradiated with the high dose of 1050 J/cm^2^. This finding is in line with observations of enhanced Ehrlich ascites tumor growth after laser irradiation which have been reported in an early paper on LLLT [12]. However it seems that typical LLLT doses ranging from 1 - 4 Joules have no influence on tumor growth, or rather they can inhibit it in implanted glioma in mice [13].

Histological data also revealed that important differences in cell morphology were induced by high doses of laser irradiation. The immune cells (lymphocytes, plasma cells and macrophages) increased in the group irradiated with the high dose of 1050 J/cm^2^. This group also presented significant areas of necrosis, a high number of atypical cells and an increase in the number of blood vessels.

Zhu et al. [8] reported differences in Focal Adhesion Kinases (FAK) and van Leeuwen et al. [7], showed differences in α-1 and β-4 subunits of integrin molecule. Both factors are important in tumor genesis and metastasis.

Many factors may contribute to tumor growth and most of them can be modulated by laser irradiation, for instance: low-level laser can enhance angiogenesis [20-22], growth factor synthesis [23-25], inflammatory metabolites [26] as well as modulate immunological cells and inflammation [27-29].

## Conclusion

LLLT administered by a dose of 150 J/cm^2 ^appears safe with only minor effects on B16F10 melanoma cells proliferation *in vitro *and no significant effect on tumor growth *in vivo*. However, a high irradiance (2.5 W/cm^2^) combined with high dose of 1050 J/cm^2^, can stimulate melanoma tumor growth with distinct histological features *in vivo*. Further studies are necessary to elucidate the main factors that are responsible for the different behaviors on tumor cells in response to laser light, and to determine laser irradiance and energy thresholds for stimulation of abnormal melanoma cell behavior.

## Competing interests

The authors declare that they have no competing interests.

## Authors' contributions

LF carried out melanoma injections in mice and histological analysis, JSSL and DAM carried out melanoma cell culture Trypan Blue dye exclusion test and MTT colorimetric test, GMF carried out of cell cycle analysis by flow cytometry and statistics, SCP and JMB were involved in drafting the manuscript and analysis, RABLM and RJB were involved in revising it critically and gave the final approval of the version to be published.

All authors have read and approved the final manuscript.

## Pre-publication history

The pre-publication history for this paper can be accessed here:

http://www.biomedcentral.com/1471-2407/9/404/prepub
